# Beyond the clinical team: evaluating the human factors-oriented training of non-clinical professionals working in healthcare contexts

**DOI:** 10.1186/s41077-019-0101-1

**Published:** 2019-06-13

**Authors:** Mary Lavelle, Gabriel B. Reedy, Chris Attoe, Thomas Simpson, Janet E. Anderson

**Affiliations:** 1 Simulation and Interactive Learning Centre, Guy’s and St. Thomas’ NHS Foundation Trust, London, UK; 2 Florence Nightingale Faculty of Nursing, Midwifery & Palliative Care, King’s College London, London, UK; 3 Faculty of Life Sciences and Medicine, King’s College London, London, UK; 4 Maudsley Simulation, London, UK; 5 School of Health Sciences, City, University of London, Myddelton Street Building, London, EC1R 1UW UK

## Abstract

**Background:**

As clinical simulation has evolved, it is increasingly used to educate staff who work in healthcare contexts (e.g. hospital administrators) or frequently encounter clinical populations as part of their work (e.g. police officers) but are not healthcare professionals. This is in recognition of the important role such individuals play in the patients’ experience of healthcare, frequently being a patients’ first point of contact with health services. The aim of the training is to improve the ability of the team to communicate and co-ordinate their actions, but there is no validated instrument to evaluate the human factors learning of non-clinical staff. Our aim was to develop, pilot and evaluate an adapted version of the Human Factors Skills for Healthcare Instrument, for non-clinical professionals.

**Method:**

The 18-item instrument was developed reflecting the human factors skills of situation awareness, decision making, communication, teamwork, leadership, care and compassion and stress and fatigue management. The instrument was piloted pre- and post-training with non-healthcare professionals (*n* = 188) attending mental health simulation training within an 11-month period (June 2017–April 2018). Trainees were hospital/primary care administrators (*n* = 53, 28%), police officers (*n* = 112, 59%), probation officers (*n* = 13, 7%) and social workers (*n* = 10, 5%). Most participants were female (*n* = 110, 59%) and from White ethnic backgrounds (*n* = 144, 77%).

**Results:**

Six items were removed, five were not sufficiently sensitive to change (*d* < .3) and one showed poor reliability. The remaining 12 items revealed a Cronbach’s alpha of .93. An exploratory factor analysis revealed a one-factor solution, which explained 58.3% of the variance. The final 12-item instrument was sensitive to change post-training (*p* < .0001) with large effect sizes (*d >* .7). Cluster analysis revealed that participants with lower pre-training scores showed the greatest improvement.

**Discussion:**

The Human Factors Skills for Healthcare Instrument-Auxiliary version (HuFSHI-A) provides a reliable and valid instrument for the evaluation of human factors skills learning following training of non-clinical populations working in healthcare contexts. Although this instrument has been developed and evaluated with training courses specifically focusing on mental health topics, HuFSHI-A is applicable for any training where teamwork and co-ordination between clinical and non-clinical professionals is considered.

## Introduction

Simulation-based training for post-graduate clinical staff is a highly effective training modality [1–3]. It usually follows the scenario-debrief training approach [4], with a debriefing style designed to scaffold the exploration of trainees’ internal beliefs and assumptions. This method is particularly effective in the learning of human factors skills, which are as essential for healthcare professionals as technical task-based skills [5–7].

Human factors skills are well defined and include situational awareness, communication, teamwork, leadership, decision making and care and compassion [8, 9]. Evaluation of the extent to which these skills are developed during simulation training is needed to ensure the effectiveness of training programmes. The Human Factors Skills for Healthcare Instrument (HuFSHI) is a validated and reliable instrument for evaluating changes in clinical learners’ confidence in their human factors skills pre- and post-training [6]. It is valid and reliable for physical and mental healthcare settings and clinically trained learners.

The delivery of patient care is a team activity. Within a hospital setting, a patient’s care journey relies on the behaviour of both clinical and non-clinical professionals including hospital porters, domestic staff and administrative and managerial staff working together to achieve common goals. Simulation training has recently begun to consider the importance of including non-clinical professionals who must interact with patients and with healthcare professionals [10, 11].

In mental healthcare in particular, non-clinical professional groups such as hospital and primary care administrators, social workers, probation officers, police officers and ambulance and hospital security staff are often the first contact for patients, particularly those experiencing deterioration in their mental health [12]. These non-clinical professionals are also involved at different stages of the processes of assessment, diagnosis and treatment and are important supports for eventual discharge into the community [13]. They are often regarded as auxiliary members of the healthcare team [14] and are frequently present at the very start of the patient’s contact with the healthcare system, a particularly critical phase as evidence suggests that negative experiences at the first contact with mental health services are associated with delays in help-seeking and resistance to treatment [15]. Simulation training is increasingly being used to educate this population about mental healthcare and effective team working in this environment [10, 15–17].

The inclusion of non-clinical professionals in healthcare simulation training courses is an important development that acknowledges the importance of good teamwork at all levels of the patient journey. Such multidisciplinary training is relevant across a range of healthcare settings where patients with physical or mental health problems present, or where clinical and non-clinical staff work together.

Evaluating the learning of human factors skills for non-clinical trainees remains challenging with no validated methods available [18, 19]. The Human Factors Skills for Healthcare Instrument (HuFSHI) provides a reliable and valid method of assessing clinical trainees’ human factors skills self-efficacy across acute and mental health settings, which is sensitive to change following training [6]. HuFSHI has been validated for use with clinical professionals, uses healthcare language and refers to clinical settings and tasks, which may be a barrier to its use with non-healthcare professionals. A further potential problem is that it was developed for degree-qualified health professionals, and so, it employs language suited to this audience and potentially difficult for others to understand. Furthermore, informal feedback from using HuFSHI with non-clinical learners indicated that the content and language were not easily understood. Therefore, the aims of this study were to:

1. Develop a new version of the Human Factors Skills for Healthcare Instrument for auxiliary healthcare (non-clinical) trainees by adapting the language used to describe human factors skills for non-clinical team members
2. Test the validity, reliability and sensitivity of the HuFSHI Auxiliary version (HuFSHI-A)
3. Identify the factor structure of the new instrument

## Methods

### Setting

The study took place in a large mental health simulation centre in South London: Maudsley Simulation at the South London and Maudsley NHS Foundation Trust. The centre provides simulation training for people working with mental health populations in community and acute settings. Ethical approval was provided by King’s College London ethics committee (RESCMR-15/16-1561).

### Participants

Participants were trainees (*n =* 188) attending simulation training at Maudsley Simulation during an 11-month period (June 2017–April 2018). They were non-clinical professionals whose job role involved contact with clinical populations and healthcare teams. They were hospital/primary care administrators (*n* = 53, 28%), police officers (*n* = 112, 59%), probation officers (*n* = 13, 7%) and social workers (*n* = 10, 5%). Most participants were female (*n* = 110, 59%) and from White ethnic backgrounds (*n* = 144, 77%).

### Item generation

The initial pool of items was drawn from the development of the HuFSHI [6], which was developed using iterative cycles of psychometric testing to choose the best items. Similarly, we anticipated that psychometric testing would identify the most effective items for this new instrument and reduce the pool of items. Therefore, to allow for this process, the initial item pool was the 18 core human factors skills items that were used to generate the HuFSHI [6]. The wording of the eighteen items was revised by psychologists [GR and ML] to be more relevant and understandable for a non-clinical audience, ensuring they reflected the same core human factor skills. During this process, the items were reviewed by non-healthcare professionals for face and content validity, readability and relevance. The stem question remained the same as in the original HuFSHI: ‘Please rate how confident you are that you can manage the following effectively’. Participants were asked to respond on a scale from 1 to 10. The 18 items from HuFSHI together with the non-clinical versions are shown in Table 1. Items in italics indicate those that were finally included in each instrument.

**Table 1:** The 18 items that were piloted in development of the original Human Factors Skills for Healthcare Instrument (HuFSHI) are displayed alongside the comparable items piloted for inclusion in the HuFSHI Auxiliary version. Items in italics are those included in each of the final 12-item instruments

Human factors skills	Human Factors Skills for Healthcare Instrument (HuFSHI) pilot items	Human Factors Skills for Healthcare Instrument-Auxiliary version (HuFSHI-A) pilot items
Care	*Constructively managing others’ negative emotions at work*	*When my colleagues are upset, I can help them calm down*
Communication/teamwork	*Requesting help from colleagues in other professions*	*I can ask colleagues from other professions for help if I need it
Communication	*Communicating effectively with a colleague with whom you disagree*	*When I disagree with a colleague, I can still work well with them
Leadership	*Prioritising when many things are happening at once*	*When many things are happening at once, I can work out what needs doing first
Teamwork	*Speaking up as part of a team to convey what you think is going on*	*I can speak up when I am part of a team to say what I think is going on*
Decision making	*Involving colleagues in your decision-making process*	*When making decisions, I can ask my colleagues for help and advice
Decision making	*Dealing with uncertainty in your decision-making process*	*When I am not sure what to do, I can still make a decision*
Situational awareness/teamwork	*Asking other team members for the information you need during a busy ward environment*	*I can ask colleagues for things I need, even if they are busy
Leadership	*Recognising when you should take on a leadership role*	*I know when I should take the lead in a team*
Situational awareness	*Monitoring the ‘big picture’ during a complex clinical situation.*	*Even when things are very busy, I can remember the goals for my team*
Situational awareness	*Anticipating what will happen next in clinical situations*	*I can think ahead, about what might happen next at work*
Teamwork	*Working effectively with a new team in clinical situations*	*I can work well with new teams I have not worked with before*
Leadership	*Re-allocating tasks between members of your team as required	*I can reassign tasks between members of my team when some people are busier than others.
Communication	*Summarising critical information for a structured handover	*I can choose the key facts I need to tell a colleague during a good handover
Decision making	*Making critical clinical decisions under pressure	*When I am under pressure, I can still make important decisions
Care	*Using effective coping strategies when experiencing stress in a clinical environment	*I can stay calm and do my job even when I am under stress.*
Communication/teamwork	*Providing constructive feedback to colleagues about their performance	*I can give my colleagues feedback that helps them do their jobs better*
Care	*Acting with compassion towards patients even when stressed	*I can show patients that I care about them, even when I am under stress.*

*Items not included in final instrument

### Procedure

Participants completed the 18-item instrument pre- and post-attending simulation training. Participants were trainees attending 11 different one-day simulation training courses, which are described in more detail in Table 2. All courses employed the scenario-debrief approach, included the important roles of non-clinical staff and contained learning objectives relating to human factors skills. All training was delivered by experienced trainers and clinical educators at the training centre.

**Table 2:** Details of the simulation training courses participants attended

Simulation course	Professions of non-clinical participants	Course overview	Course learning objectives	Example scenario
Police and ambulance service – mental health awareness	Police officers	This course aims to equip police and ambulance staff with the skills required to support people with mental health needs, as they often present initially to these professions	- Improved confidence, knowledge and skills in recognising, assessing and managing people with mental health conditions - Enhanced understanding of the role of human factors in supporting people with mental health conditions - Increased communication and collaboration skills with multi-disciplinary professionals	A police officer has been called to investigate disturbances at a property reported by a neighbour, where they are required to risk assess someone with suicidal ideation
Managing mental health situations for non-clinical staff	Administrators	This course aims to support non-clinical staff to develop skills that may help them manage challenging situations in the workplace	- Improved interactions with service users and family members, reflecting on their experiences - Enhanced confidence when communicating with distressed and agitated service users and relatives - Increased understanding of how to manage difficult conversations with colleagues	You receive a phone call from a 30-year-old female known to your team who reports that she is distressed and contemplating suicide
Primary care navigator skills	Administrators (primary care)	This course aims to support pre-identified non-clinical staff assume the role of primary care navigators to help coordinate patients’ care	- Improved confidence to interact with service users and family members and liaising with professionals - Enhanced confidence when communicating with distressed and agitated service users and carers - Increased ability to empathise with and reflect on service users’ experiences	A carer of his frail elderly mother arrives in the surgery unhappy with the care his mother is receiving, subsequently reporting that he has ‘sacked’ her carers
Mental health workshop for primary care administrators	Administrators (primary care)	This course aims to develop the skills and understanding required to support people with mental health conditions in primary care	- Improved understanding of human factors in supporting people with mental health needs - Enhanced knowledge and recognition of common mental health conditions - Increased confidence in approaching and providing support to distressed and agitated patients	An elderly patient with dementia has arrived at the surgery confused and worried about where he is and why he is there, as he becomes increasingly anxious
Mental health awareness for probation officers	Probation officers	This course aims to build probation workers’ ability to working with people experiencing mental health conditions across a variety of situations	- Improved skills to manage difficult situations and engagement with service users - Increased collaboration and communication skills with statutory, community and health services - Enhanced use of human factors skills in supporting people with mental health needs	Home visit to see a 35-year-old male recently released from prison following breach of a suspended sentence order for drunk driving, raising safeguarding concerns relating to his pregnant girlfriend
Early intervention and prevention in children’s health	Administrators and support workers	This course aims to support those working with young people and families in primary care to intervene early in mental illness and promote wellbeing	- Improved recognition of risks and signs of mental illness in young people - Enhanced confidence in risk assessing and intervening in mental health needs - Increased skills in working with mental and physical health, emotional distress, and families and carers	Tanya, a 15-year-old school girl, is struggling with somatic symptoms and depression related to cyber-bullying noted by her school nurse; however, her mother is dismissive of the problems
‘Starting the conversation’: end of life care	Support workers and social workers	This course aims to empower health and social care staff to start end of life care conversations and advanced care planning at the right time for patients and families	- Improved communication skills with people with dementia and their carers about sensitive issues - Enhanced knowledge of best practice in having early conversations about end of life decisions and planning - Increased confidence in having these conversations and addressing various needs across the care pathway	Peggy has vascular dementia following a stroke. You are asked to have a planning conversation with Peggy in her home with her main carer and foster daughter Carol
Perinatal mental health	Social workers	This course aims to bring together professionals from all settings of health and social care that may support mothers, babies and families with mental health needs during the perinatal period	- Improved confidence, knowledge and skills in assessing and managing perinatal mental illness - Greater confidence in undertaking comprehensive risk assessments of perinatal mental illness Enhanced understanding of collaborating with the range of agencies involved in perinatal health	A mother, her baby and partner are on the obstetric ward about medical complications at birth, with the mother becoming increasingly agitated and showing signs of postpartum psychosis
Opportunistic interventions for alcohol and drugs	Social workers	This course aims to support healthcare staff to provide service users with brief interventions for alcohol and drug use when the opportunity arises	- Improved ability to screen for and identify harmful use of substances - Enhanced ability to deliver a brief intervention in a sensitive and non-judgemental way - Increased knowledge of alcohol and substance abuse, local services and resources	A woman in her 50s has presented to Accident & Emergency with a head laceration and has now been cleared for discharge, the team suspect that she has been intoxicated and screening has indicated hazardous drinking

Participants were informed about the study both verbally and through a participant information sheet. The instrument was completed by consenting participants at the start of the training day (pre-training) and at the end of the training day (post-training).

### Statistical analysis

#### Item selection

Initial analyses on the first pool of 18 items were conducted using IBM SPSS (V.24) [20]. Evaluation of the items was achieved in four steps:

1. Participant responses to each item were examined descriptively to identify ceiling and floor effects.
2. As sensitivity to change pre- and post-training was a critical feature of the instrument, paired samples *t* tests assessed the change in item scores pre- to post-training, Cohen’s *d* effect sizes were calculated for each item and items with a small effect size (*d* < .3) were eliminated from the instrument. This is standard practice in item selection and instrument development: multiple items representing the same construct are proposed and tested and the items that do show sensitivity to change would be retained while those that are not are eliminated [21].
3. Inter-item correlations were examined to assess for redundancy between items, while balancing with theoretical justifications for item selection.
4. An exploratory factor analysis (EFA) using a maximum likelihood factor extraction method was conducted. Only factors with Eigenvalues over 1 were extracted.

#### Instrument sensitivity to change

The sensitivity of the final instrument was explored in a further two steps:

5. Paired samples *t* tests compared pre- and post-training scores for the final instrument for the whole sample and for the two most common professional groups (administrators and police).
6. The HuFSHI Clinical Version compared change pre- and post-training for experienced and novice healthcare professionals based on their years of clinical experience. However, due to the diversity in professional groups in our sample, experience was not deemed to be an appropriate index. Instead, Hierarchical Cluster Analysis explored characteristics of groups of participants based on their pre- and post-training scores. A Ward’s cluster method was employed with a squared Euclidean distance method measuring distance between cases. This method combines individual cases into clusters. The relative distance between clusters informed the number of distinct clusters that were present. The difference between clusters in terms of participant characteristics and instrument scores was compared using appropriate inferential statistics.

## Results

### Item selection

#### Step 1

No ceiling or floor effects were observed for the 18 items with all displaying normal distributions and skewness within normal levels (range − 1.7 to − 0.59).

#### Step 2

Paired samples *t* test assessed the change in item scores pre- to post-course, and Cohen’s *d* effect sizes were calculated for each item. Nine participants were excluded from this analysis as they did not complete the post-course questionnaires. Five items with small effect sizes (*d* < .3) were identified and removed: item 2—I can ask colleagues from other professions for help if I need it; item 3*—*When I disagree with a colleague, I can still work well with them; item 4*—*When many things are happening at once, I can work out what needs doing first; item 6*—*When making decisions, I can ask my colleagues for help and advice; item 8*—*I can ask colleagues for things I need, even if they are busy. The number of items after this step was 13.

The items eliminated in this step may have shown small effect sizes due to a number of potential factors including item phrasing, wording, or lack of conceptual clarity to participants. However, the human factors skills that these items referred to were also represented by other items that were retained in the item pool. Thus, although these specific items were not sensitive to change, it does not mean that the skills they represent did not improve, but merely that these items were not the most effective items for detecting these changes [21].

#### Step 3

Inter-item correlations for the remaining 13 items were conducted on the pre-course questionnaires and are displayed in Table 3. Reliability analysis of these 13 items revealed a Cronbach’s alpha of .93. All items were significantly positively correlated with item-total correlations ranging from *r =* .61 to *r =* .80. Two items showed high item-total correlations of *r =* .80 (item 14—I can choose the key facts I need to tell a colleague during a good handover and item 15*—*When I am under pressure, I can still make important decisions). Despite the high item-total correlations, these items were retained to ensure that the six human factors skills (situation awareness, decision making, communication, teamwork, leadership and care) were adequately represented in the final tool.

**Table 3:** Pearson’s correlations between items alongside for each item: the item total correlation, Cronbach’s alpha if deleted and the exploratory factor analysis factor loadings

Human factors skills	Items	5	7	9	10	11	12	13	14	15	16	17	18	13 item set alpha = .936		Final 12 item instrument alpha = .934		
														Corrected item-total correlations	Cronbach’s alpha if item deleted	Corrected item-total correlations	Cronbach’s alpha if item deleted	Factor loadings EFA
Care	1. When my colleagues are upset, I can help them calm down	.523	412	.508	.446	.377	.452	.544	.465	.514	.533	.528	.484	.64	.932	.63	.930	.644
Teamwork/communication	5. I can speak up when I am part of a team to say what I think is going on		.465	.615	.618	.503	.513	.541	.607	.604	.525	.500	.427	.71	.930	.71	.927	.733
Decision making	7. When I am not sure what to do, I can still make a decision			.478	.499	.505	.473	.590	.536	.589	.525	.470	.378	.65	.932	.65	.929	.675
Leadership	9. I know when I should take the lead in a team				.592	.428	.463	.641	.665	.607	.603	.633	.506	.74	.928	.74	.926	.771
Situational awareness	10. Even when things are very busy, I can remember the goals for my team					.552	.494	.544	.638	.612	.569	.486	.447	.72	.929	.72	.927	.745
Situational awareness	11. I can think ahead, about what might happen next at work						.514	.579	.531	.564	.414	.401	.290	.62	.932	.63	.930	.650
Teamwork	12. I can work well with new teams I have not worked with before							.517	.487	.481	.442	.459	.382	.62	.932	.62	.930	.631
Leadership	13. I can reassign tasks between members of my team when some people are busier than others.								.615	.571	.549	.662	.397	.75	.928	.75	.925	.766
Communication	14. I can choose the key facts I need to tell a colleague during a good handover									.779	.667	.664	.538	.80	.927	.79	.924	.844
Decision making	15. When I am under pressure, I can still make important decisions										.712	.607	.559	.80	.927	.79	.924	.837
Care	16. I can stay calm and do my job even when I am under stress.											.624	.610	.74	.929	.74	.926	.775
Teamwork/communication	17. I can give my colleagues feedback that helps them do their jobs better												.586	.73	.929	.72	.927	.755
Care	*18. I can show others that I care, even when I am under stress.												–	.61	.933	–	–	–

*Items excluded prior to EFA. Not included in the final instrument

Item 18 (I can show others that I care, even when I am under stress) was one of the three items representing the human factors skills of care. This item had the lowest item-total correlation (*r* = .61), and deleting it had no impact on the Cronbach’s alpha; therefore, it was removed from the final instrument to reduce redundancy. Twelve items were retained after this step.

#### Step 4

Reliability analysis on the remaining 12 items revealed a Cronbach’s alpha of .934. An exploratory factor analysis of pre-training scores was conducted using a maximum likelihood method extracting factors with Eigenvalues greater than 1. The Kaiser-Meyer-Olkin measure of sampling adequacy was .94, and the Bartlett’s test of sphericity was highly significant (chi-squared = 1399.29, df = 44, *p* < .0001).

The Scree plot produced in the exploratory factor analysis revealed a one-factor solution that explained 58.3% of the variance. The factor loadings of each item are displayed in Table 3 (range .63 to .84).

### Sensitivity to change

#### Step 5

Comparing mean scores for the final 12-item instrument pre- and post-training revealed that participants’ scores significantly improved post-training (*p* < .0001) overall and at the professional group level (*p < .*001) with large effect sizes (*d > .*7) (see Table 4).

**Table 4:** Paired samples *t* test comparisons of mean 12-item instrument scores by professional group

	Pre-training *M* (SD)	Post-raining *M* (SD)	*t*	df	*p*	*d*
All participants	7.80 (1.1)	8.40 (1.0)	10.81	177	< .001	.82
Administrators	7.63 (1.5)	8.40 (1.2)	7.48	49	< .001	.88
Police	7.97 (1.0)	8.46 (0.9)	7.51	110	< .001	.88

#### Step 6

**Fig. 1:**
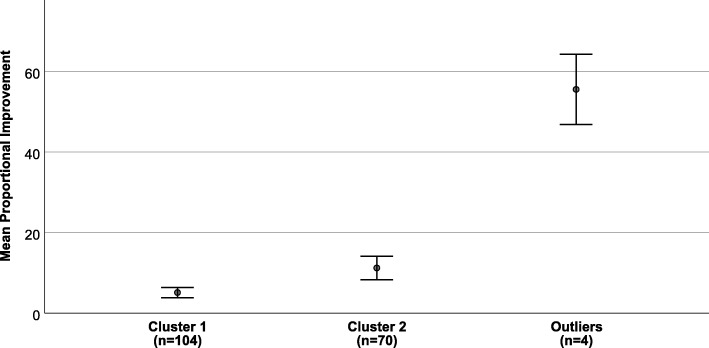
Mean proportional improvement of participants scores post-training by identified clusters alongside participants identified as outliers

Participants’ pre- and post-scores of the final 12-item instrument were entered into the cluster analysis. Four participants were further excluded as they were identified as outliers in the cluster analysis process; these cases are described in more detail below (see the “Outliers” section). After exclusion of these cases, the final dendrogram revealed two clear clusters, cluster 1 contained 104 cases, while cluster 2 contained 70 cases. The chi-squared analysis revealed that clusters did not differ significantly in terms of participant characteristics (Table 5). Independent samples *t* test revealed that participants in cluster 1 had significantly higher pre- and post-training scores, compared to participants in cluster 2. The proportional improvement score was calculated for each participant as the difference between pre- and post-training scores, divided by the pre-training score and multiplied by 100 (i.e. [((post-score-pre-score)/pre-score) × 100]). The proportional improvement scores were significantly greater for participants in cluster 2 (*M* = 11.23, SD = 12.28; range − 18 to 43) compared to cluster 1 (*M* = 5.11, SD = 6.54; range − 10 to 27) (*z* = 3.23, *p* = .001) (see Fig. 1). Following psychometric testing, the final instrument contained 12 items.

**Table 5:** Comparisons of instrument scores and participant characteristics by identified clusters

	Cluster 1 *n* = 104 *M* (SD)	Cluster 2 *n* = 70 *M* (SD)	*t/χ* ^*2*^	df	*p*
Instrument scores					
Pre-training	8.58 (.65)	6.85 (.62)	17.33	174	< .0001
Post-training	9.02 (.66)	7.65 (.68)	13.31	174	< .0001
Participant characteristics					
Years qualified	3.22 (6.9)	3.05 (5.42)	.17	174	.86
Age (%)			1.87	3	.60
< 25	10	17			
25–34	32	33			
35–44	43	41			
45–55	13	9			
% female	42	44	.93	1	.34
% White	78	75	.02	1	.89
% qualified	71	75	.59	1	.74
Professional group (%)			5.05	3	.17
Administrators	28	26			
Police	65	61			
Probation officers	6	9			
Social workers	2	4			
% who actively participated in a scenario during training	100	98	.87	1	.35

### Outliers

The four outliers identified comprised of two administrators and two members of the police. The pre- and post-training scores of these participants were particularly low (pre-training scores *M* = 5.15, SD = .97; range 3.90–6.30; post-training scores 7.52, SD = 1.35; range 5.90–9.00). However, the proportional improvement of these participants was greater than the upper limits of participants in either clusters 1 or 2 (*M* = 55.5, SD = 5.4; range 50–61) (see Fig. 1).

## Discussion

The aim of this study was to develop an instrument to evaluate the learning of human factors skills in non-clinical staff working in healthcare settings, or with clinical populations. The new instrument, titled the Human Factors Skills for Healthcare Instrument-Auxiliary version (HuFSHI-A), is a 12-item instrument with a single factor structure. It is reliable, with face and content validity and sensitive to change post-training. This is the first instrument that has been specifically developed for use with non-clinical populations receiving simulation training in the context of healthcare. This will enable better design and evaluation of simulation training for non-clinical populations.

This instrument has been developed and validated in a healthcare educational setting in which the training focused on human factors skills in a mental healthcare context. Although the courses differed in content, a focus on improving participants’ human factors skills in scenarios relevant to their daily work was maintained throughout the development, delivery and review of all courses. Human factors skills were embedded in the course learning outcomes, the scenario design and the debrief approach, and training was delivered by experienced trainers. Therefore, it would be expected that participants’ human factors skills self-efficacy would improve following such training, providing a rationale for evaluating the instrument in this robust training environment. The diversity of course learning objectives (Table 2) across nine different training courses, alongside the diversity of the participants’ professional backgrounds (primary and secondary care administrators, police officers, probation officers and social workers), provides reassurance that the final instrument is widely applicable.

Data gathered during the instrument development phase showed that non-clinical trainees’ self-efficacy in their human factors skills increased significantly post-training and improved more for those whose pre-course scores were low. This pattern of low pre-training scores and higher improvement scores was particularly pronounced for four trainees in this cohort who were identified as outliers. Participants’ pre-training scores and improvement scores were not associated with their professional group, years qualified or any demographic characteristics. For trainees with lower pre-training scores, human factors skills may have been a concept they had not encountered prior to the training. This study provides evidence that exposure to and practice of these skills through simulation training leads to significant improvements especially when participants are less confident to begin with. If non-clinical populations clearly benefit from simulation training, there is a clear rationale for including them in training programmes.

HuFSHI-A has both face and content validity [22]. Due to a lack of available tests of this type, it was not possible to measure criterion validity at this stage [22]. A contemporary approach to validity is addressed through Kane’s framework [23], which examines an instrument’s validity in the context of its specific purpose. It is comprised of four steps: scoring, generalisation, extrapolation and implication. Kane’s framework focuses on the development of instruments to be used in assessment decisions. As such, it emphasises the implications of assessment tools, instruments and approaches focused on measuring individual attainment for the purposes of admission, progression or award decisions. The HuFSHI-A is not an assessment tool; its purpose is to help educators to determine the extent to which simulation training is effective at improving non-clinical learners’ self-efficacy around human factors-oriented skills. It is not intended for the purposes of individual assessment. Therefore, extrapolating from individual scores to real-world performance (step three in Kane’s framework) would not be an appropriate validation of this instrument (although previous studies have shown that similar self-efficacy measures do correlate with work-related performance [24]). Similarly, Kane’s final validation step ‘Implication’ evaluates the consequences or impact of the assessment on the learner, whereas HuFSHI-A has no implications for individual learners. Rather, the results would, we argue, help to inform decisions regarding training design and delivery.

Despite some lack of fit between Kane’s framework and the purpose of the current instrument, HuFHSI-A does meet Kane’s first two steps of validity (i.e. scoring and generalisation). Scoring validity is evidenced by rigorous item selection procedures [6] and use of item scoring which is consistent with theory and practice in self-efficacy instrument design [21]. Generalisation validity is evidenced by diversity in the pilot data, both in terms of training content and trainee professional groups. Thus, we claim that this instrument has a valid scoring procedure and is generalisable to the populations with which it was designed (i.e. non-clinical professionals working in healthcare settings, or with clinical populations). We do not claim that this instrument has validity beyond this context; however, we believe this context is sufficiently broad to be confident in its use in similar settings for the purposes of evaluating and informing simulation training.

The clinical training content in the current sample focused on mental health. However, the human factors-oriented learning objectives of the training courses, which were the focus of this evaluation instrument, were not specific to mental health but are general human factors skills (i.e. situational awareness, communication, teamwork, leadership, decision making and care and compassion), which traverse all aspects of working within clinical contexts, irrelevant of the nature of the clinical situation (e.g. mental or physical health). As such, the clinical topics of the training courses provide the context for communicating these topics, but the human factors skills themselves are not bound to any clinical situation: they are transferable across all aspects of team working in a healthcare context. For this reason, we anticipate that the HuFSHI-A would be applicable for evaluation of human factors skills learning following educational training in any context where clinical and non-healthcare professionals work together.

Joint training of clinical and non-clinical staff has received little attention but is increasingly necessary as non-healthcare professionals are recognised as some of the first-line contacts in patient care [12]. Mental healthcare simulation programmes are leading the development of such multi-disciplinary training programmes because this is where the need is greatest [10, 25]. However, human factors skills are the building blocks of effective communication and team working. Effective patient care in any healthcare context relies on effective teamwork, not just between healthcare professionals but also with non-clinical team members, e.g. administrators, receptionists and managers. Improving human factors skills in both clinical and non-clinical staff working in any healthcare context has the potential to improve the efficiency and effectiveness of team working, leading to improvements in the provision of healthcare more broadly. Joint training of clinical and non-clinical staff is therefore an important consideration across all healthcare sectors. The provision of tools such as HuFSHI and HuFSHI-A to evaluate learning following training is a step towards broadening this practice.

## Conclusions

Simulation training develops human factors skills that are essential for clear communication and good teamwork. Although there are instruments available to evaluate learning following simulation, these are not tailored to non-healthcare professionals who are increasingly being included in training programmes. The strengths of this instrument are that it was empirically developed, has good validity and reliability and is brief and therefore feasible to incorporate into busy training programmes. Its use will facilitate the development of effective training human factors programmes and effective teamwork between clinical and non-clinical disciplines. Further research could examine its application alongside training in other healthcare settings.

## Data Availability

An online version of the Human Factors Skills for Healthcare Instrument – Non-Clinical Version is available from the authors upon request. For more information, please contact HuFSHI@kcl.ac.uk.

## Abbreviations

HuFSHI: Human Factors Skills for Healthcare Instrument
HuFSHI-A: Human Factors Skills for Healthcare Instrument-Auxiliary version
